# Ultrasound Effect on the Microstructure and Hardness of AlMg3 Alloy under Upsetting

**DOI:** 10.3390/ma14041010

**Published:** 2021-02-20

**Authors:** Przemysław Snopiński, Tibor Donič, Tomasz Tański, Krzysztof Matus, Branislav Hadzima, Ronald Bastovansky

**Affiliations:** 1 Department of Engineering Materials and Biomaterials, Silesian University of Technology, 18A Konarskiego Street, 44-100 Gliwice, Poland; Tomasz.tanski@polsl.pl; 2 Research Centre of University of Žilina, University of Žilina, 010 26 Žilina, Slovakia; tibor.donic@gmail.com (T.D.); branislav.hadzima@rc.uniza.sk (B.H.); 3 Materials Research Laboratory, Silesian University of Technology, 18A Konarskiego Street, 44-100 Gliwice, Poland; krzysztof.matus@polsl.pl; 4 Department of Design and Mechanical Elements, University of Žilina, Univerzitná 8215/1, 010 26 Žilina, Slovakia; ronald.bastovansky@gmail.com

**Keywords:** Al–Mg alloy, ultrasonic vibrations, upsetting, structure, hardness

## Abstract

To date, numerous investigations have shown the beneficial effect of ultrasonic vibration-assisted forming technology due to its influence on the forming load, flow stress, friction condition reduction and the increase of the metal forming limit. Although the immediate occurring force and mean stress reduction are known phenomena, the underlying effects of ultrasonic-based material softening remain an object of current research. Therefore, in this article, we investigate the effect of upsetting with and without the ultrasonic vibrations (USV) on the evolution of the microstructure, stress relaxation and hardness of the AlMg3 aluminum alloy. To understand the process physics, after the UAC (ultrasonic assisted compression), the microstructures of the samples were analyzed by light and electron microscopy, including the orientation imaging via electron backscatter diffraction. According to the test result, it is found that ultrasonic vibration can reduce flow stress during the ultrasonic-assisted compression (UAC) process for the investigated aluminum–magnesium alloy due to the acoustic softening effect. By comparing the microstructures of samples compressed with and without simultaneous application of ultrasonic vibrations, the enhanced shear banding and grain rotation were found to be responsible for grain refinement enhancement. The coupled action of the ultrasonic vibrations and plastic deformation decreased the grains of AlMg3 alloy from ~270 μm to ~1.52 μm, which has resulted in a hardness enhancement of UAC processed sample to about 117 HV.

## 1. Introduction

The emission of greenhouse gases caused by human activities is one cause of climate change. The emissions derived from the transportation sector worldwide have largely increased over the last decades. This sector actually contributes to almost a quarter of the global carbon dioxide (CO_2_) emissions. Most of them come from passenger vehicles—cars and buses—due to the fossil fuels (diesel/petrol) burned on the road [1,2].

In order to decrease the greenhouse gas emissions, the European community policies defined the targets for the future emissions of the new cars—the European car fleet should be reduced—stepwise to 65 g CO_2_/km by 2030 [3]. As these regulations have been strengthened, many studies have been conducted to improve the efficiency of automobiles.

A promising and efficient way to achieve the greenhouse gas emission reduction is the application of lightweight materials [4,5,6]. Typical materials, such as carbon steel and cast iron, can be replaced by materials with a higher strength to density ratio. It is reported that each kilogram of aluminum that replaces mild steel, high strength steel and cast iron avoids 13–20 kg of greenhouse gas emissions [7]. Weight reduction provides an improvement in terms of vehicle technical features and performances, such as higher speed and acceleration [8]. At the same time, the lower mass means better stability and handling as well as a shorter braking distance, thus providing a substantial contribution to meet the continuously rising legal safety requirements [9,10].

Among the different series of aluminum alloys, the role of the 5xxx series with magnesium as the main alloying element is vital. The aluminum–magnesium alloys are a potential candidate for many structural applications in the automotive industry because of their reasonable strength, high corrosion resistance, low alloying content and single-phase structure (free of precipitates) [11,12,13,14,15] However, the most serious deficiency of the Al–Mg alloys with the Mg content ≥ 3 wt% is poor technological plasticity. Therefore, to date, many studies have been conducted to increase the formability of the Al–Mg alloys. It demonstrated, for example, that the addition of Sc or Zr [16,17] as well as pre-aging [18] improves the formability of the Al–Mg alloys. Multiple studies have also shown that the application of ultrasonic vibrations (USV) leads to decreasing the deforming resistance and increasing the formability [19,20,21]. This is caused by the fact the ultrasonic vibration causes a stress superposition and acoustic softening during deformation, which significantly reduces the yield strength [22]. However, the effects of the ultrasonic vibrations are more complex. Pal et al. [23] illustrated that ultrasonic vibration could increase the dislocation density. It has also been demonstrated that the ultrasonic vibrations are capable of refining the grain size and thus improving the mechanical strength of the samples [24,25]. The investigation of Liu et al. [26] indicated that the ultrasonic wave during the upsetting process leads to fabricating the UFG structure on the pure copper cone tips. Although multiple studies have shown a beneficial effect of the USV on the structure and mechanical properties [27,28,29], the detailed research concerning vibration impact on the material formability as well as grain refinement in the Al–Mg alloy system is limited. To date, we found only one publication related to the application of USV [30] in the Al–Mg alloy system and a few related to the Al–Mg–Si system [31,32,33].

Therefore, in this article, the ultrasonic vibration-assisted upsetting and conventional upsetting processes were carried out for a comparative study of the extrapolated compression tests on the Al–Mg alloy samples to obtain the stress–strain relation under ultrasonic-vibration. Through observing the microstructure of the samples obtained from these two forming processes using an optical microscope and transmission electron microscope, the effect of the ultrasonic vibration on the microstructure of the specimens during the plastic deformation process is discussed.

## 2. Materials and Methods

**Figure 1:**
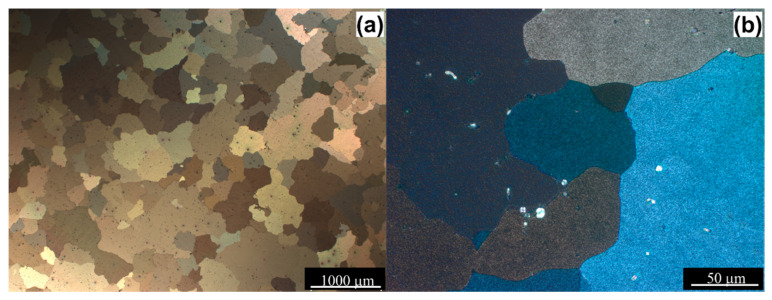
Microstructure of the AlMg3 alloy in T6 conditions. (**a**) low magnification image; (**b**) high magnification image.

The material used in this study was the AlMg3 aluminum alloy, of which the measured chemical composition of the metal is given in Table 1, and the initial microstructure of the AlMg3 alloy is presented in Figure 1. Prior to deformation, specimens were first solid solution treated at 853 K for 8 h, followed by quenching in water and artificially aged at 433 K for 8 h (T6) [34].

**Figure 2:**
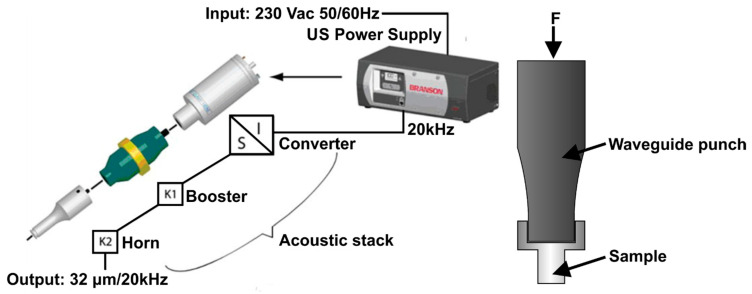
The main components of the experimental setup used in this study.

The upsetting of the material was conducted at room temperature using the Emerson-Branson system under a load of 4  kW. The compressive deformation was implemented both with the ultrasonic excitation and without it at a constant cross-head speed of 5 mm/min. The generation of ultrasonic vibrations (USV) was conducted using a Branson 4.00DCXs20VRT power supply (Branson, Nove Mesto nad Vahom, Slovakia). The application of USV to the Al–Mg sample in the process of compressive deformation was conducted using an acoustic stack comprising a converter, a booster and a horn. The experimental sample was installed on the butt end of the horn (Figure 2). The power of ultrasound was at the end of the horn, and the ultrasonic device was able to operate in the interval of power 0–4 kW. The axial amplitude of USV on the end of the horn was 32 µm.

The following conditions were produced, differing in terms of the deformation regimes:

- condition 1—upsetting by 80% without the ultrasonic excitation;
- condition 2—upsetting by 80% with the ultrasonic excitation. In this condition, ultrasonic excitation was applied for two intervals. First, at an ε = 0.03 for 12 s, then discontinued and, after 18 s of continued static compression, was applied again for 12 s.

**Figure 3:**
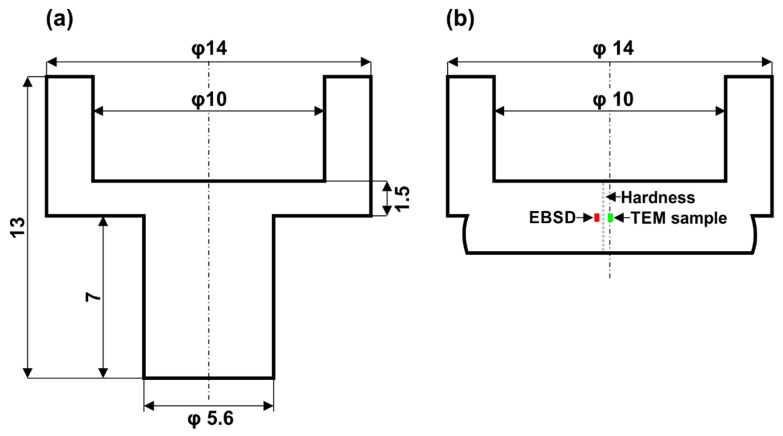
Dimensions of the sample (**a**) prior deformation; (**b**) after deformation.

The detailed dimensions of the sample prior and after upsetting are shown in Figure 3.

The deformed samples were then sectioned along the compression axis for a microstructural observation carried out by light, electron backscattered diffraction (EBSD) (Carl Zeiss NTS GmbH, Oberkochen, Germany) and transmission electron microscopy (TEM) (Jeol, Tokyo, Japan). The samples for EBSD were mechanically polished following the standard metallographic procedures and then electro-polished using the Struers A2 electrolyte (Struers, Westlake Cleveland, OH, USA). Since the microstructure obtained after the compression tests was different depending on its position within a sample, the EBSD analysis was conducted at a position that was 1/2 away from the surface toward the center of the sample (Figure 3b). Scans were conducted on the Zeiss Supra 35 scanning electron microscope (Carl Zeiss NTS GmbH, Oberkochen, Germany) equipped with the EBSD detector and TSL OIM software (EDAX, Inc., Mahwah, NJ, USA). An area of 150 × 180 μm^2^ was scanned, and the step size was taken to be 0.35 μm in all scans. The collected data were subjected to a standard clean-up procedure consisting of (i) grain dilation with GTA of 5° and minimum grain size of 2 pixels; (ii) grain confidence index (CI) standardization with GTA of 5° and minimum grain size of 2 pixels; (iii) neighbor orientation correlation (level 4) with a minimum CI of 0.02. Then the EBSD data were finally post-processed using the Atex software [35].

A focused ion beam (FIB) technique was used to prepare the TEM samples. For this purpose, samples were cut towards the compression direction at a position that was 1/2 away from the surface toward the center of the sample. Samples were then analyzed in the transmission electron microscope JEM 3010UHR from JEOL at an accelerating voltage of 200 kV.

Finally, the Vickers microhardness (HV) of the samples was measured on the electro-polished surfaces. The indentations were made with a load of 100 gf and a dwell time of 15 s using an FM ARS 7000 hardness tester. Measurements were taken along the thickness of the sample with the average distance between individual indentation points of 0.2 mm.

## 3. Results

### 3.1. Light Microscopy

**Figure 4:**
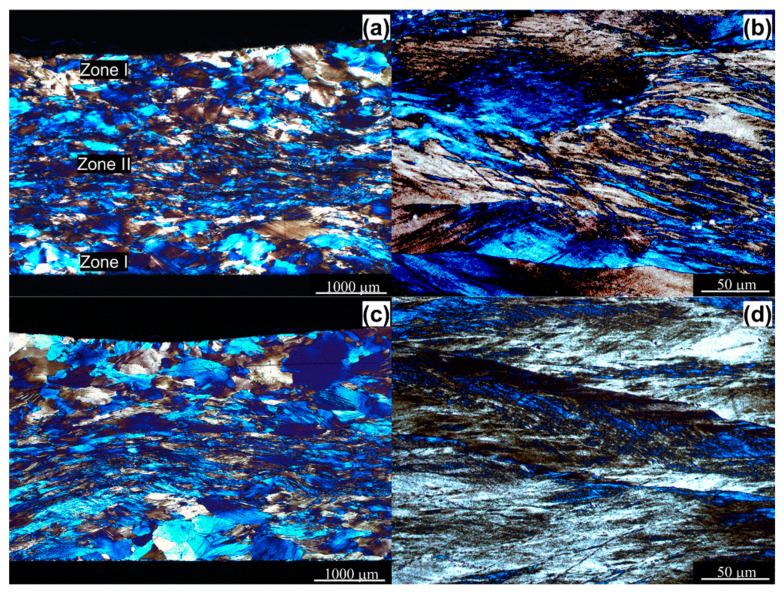
Microstructure of the AlMg3 aluminum alloy (**a**,**b**) condition 1 (without ultrasonic vibrations), (**c**,**d**) condition 2 (with ultrasonic vibrations).

Figure 4 shows the details of the microstructure after upsetting. From these images, it is possible to determine the most intensive directions of the metal flow along the diagonal direction of the compressed sample area. The deformed sample microstructure can be divided into two zones according to the observed degree of deformation. Among these two zones, the deformation degree of zone I (near the top and bottom of the samples) is the smallest—the plastic flow of metal in this zone was limited by the friction effect between the end surfaces and waveguide punch or a bottom barrel, while the deformation degree of the zone II (in the middle of the samples) is the largest one (Figure 4a,c). The observations under higher magnification (Figure 4b,d) of zone II (affected by a higher amount of strain) reveal a high degree of microstructure refinement. The initial coarse-grained microstructure (Figure 1) became remarkably elongated perpendicularly to the compression direction and refined through shear banding. By direct comparison of both images (Figure 4b,d), it appears that the density of the macroscopic shear/deformation bands for the sample deformed by the action of the USV is greater in comparison with the sample deformed without USV.

### 3.2. EBSD Analysis

**Figure 5:**
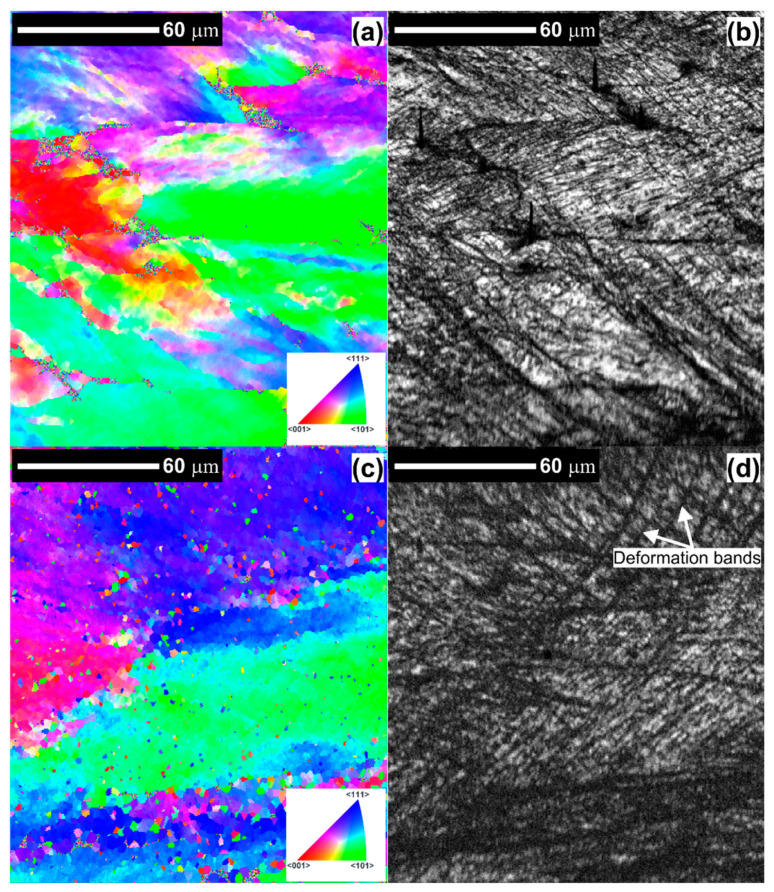
Electron backscattered diffraction (EBSD) maps of the AlMg3 alloy (**a**,**b**) upsetting without USV, (**c**,**d**) upsetting with USV.

Figure 5a–d shows the typical inverse pole figure (IPF) and image quality (IQ) EBSD maps of the AlMg3 alloy subjected to upsetting with and without ultrasonic excitation. The visible dark patches in the image quality maps (black areas) results from the low band contrast indicate highly strained regions composed of shear/deformation bands, which is in agreement with our light microscopy analysis. As can be observed, the deformation-induced shear band formation has resulted in significant grain refinement. According to the study of Madhavan [36], these shear bands can be the Cu type (since they are generally developed within the initial coarse grains), which is commonly observed in the high- and medium-SFE materials.

The microstructures of both samples are composed of a mixture of elongated grains and mutually crossing deformation bands (Figure 5b–d). By the careful analysis and comparison of the (IQ) EBSD maps, it can be proved that the fraction of shear bands (typically required for relaxing stress concentration during deformation) is much greater in the microstructure of the sample deformed with ultrasonic excitation. Comparing the IQ maps to the IPF maps, we observe that the darker areas in the IQ images (shear bands) contain many small grains that are separated by HAGBs.

**Figure 6:**
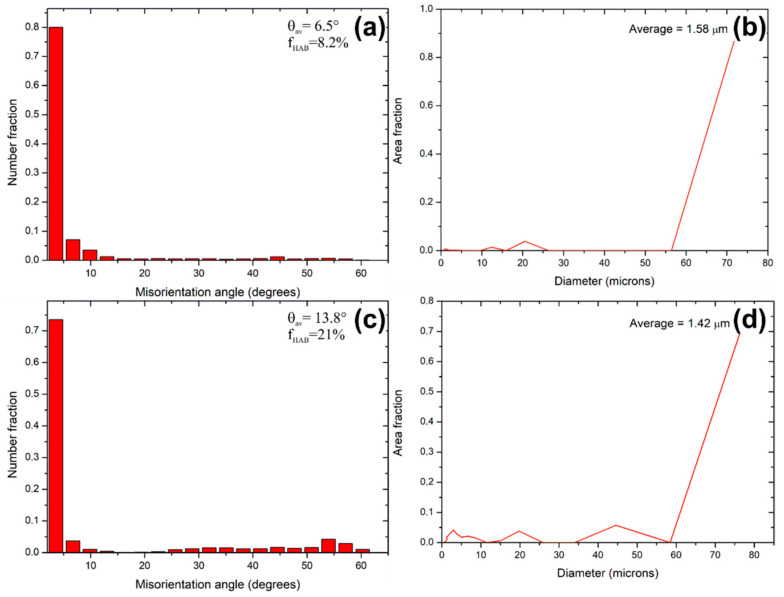
Grain size distribution and misorientation maps of AlMg3 alloy (**a**,**b**) upsetting without ultrasonic vibrations (USV), (**c**,**d**) upsetting with USV.

The grain size distributions and misorientation maps obtained from the EBSD analysis are shown in Figure 6. As can be observed, the fraction of low-angle grain boundaries is higher than the high-angle boundaries, suggesting the significant generation of dislocations after compression, Figure 6a,c. From these images, it is also easily seen that the application of USV during compression gives rise to a higher fraction of high angle boundaries (HAGBs; ~21%) so that the average grain boundary misorientations θ_av_ increase to 13.8°. Our observation does not coincide with these literature reports [25,37,38], where researchers observed an opposite effect. However, in these articles, the ultrasonic excitation time is short—typically a few seconds, as well as the total applied strain is much lower. According to the article of Hu et al. [37], at small deformation strain, ultrasonic-induced dynamic recovery promotes dislocation annihilation, leading to the dislocation density reduction, which hinders sub-grain formation. Here the reason for the increase in the fraction of HAGBs after USV deformation could be increased dislocation density (due to long excitation time) and their mobility due to the oscillatory stress wave [39]. According to this research [28], ultrasonic vibrations facilitate the transmission of dislocations through grain boundaries, their incorporation into grain boundaries, and other processes of rearrangements, which can lead to a change of misorientations of the boundaries and grain rotation. As a result, smaller grains with higher misorientations can be obtained. Aside from this mechanism, the shear banding might have a meaningful influence on the fraction of high angle grain boundaries. According to the work of Bakai et al. [40], the ultrasonic vibrations may initiate the formation of shear bands. As a result of intensified shear banding, coarser grains become divided into areas having diversified misorientations—usually high angle misorientation.

### 3.3. TEM Analysis

**Figure 7:**
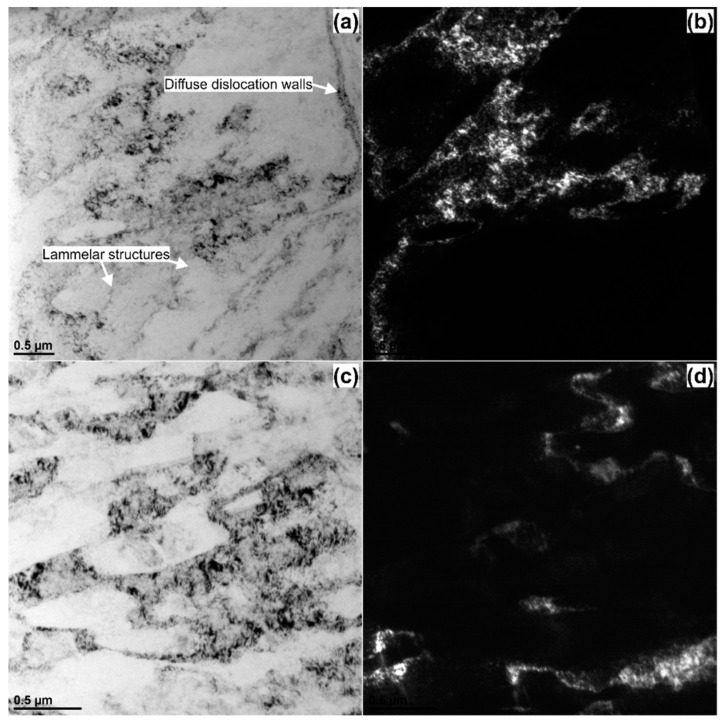
Bright and dark field TEM images of the AlMg3 alloy (**a**,**b**) upsetting without USV, (**c**,**d**) upsetting with USV.

Figure 7 shows bright and dark field TEM images comparing the morphology of the sub-micron structure of the AlMg3 samples after upsetting without ultrasonic vibrations a–b and with ultrasonic vibrations c–d. As can be seen, both images show very diversified microstructures. The observations made via transmission electron microscope (TEM) revealed that compression without any USV fabricated typical dislocation structures, Figure 7a. The microstructure consists of (i) dense dislocation areas (diffuse dislocation walls) as well as (ii) cleaner ones containing coarse lamellar structures. This microstructure has a visual appearance typical for the Al–Mg alloys subjected to low/medium strain deformation ε < 2 [41]. In contrast, in the microstructure of the sample deformed by the ultrasonic vibrations, Figure 7c,d, parallel bands, well-defined cells and subgrains with a high diffraction contrast at the boundaries can be observed. It is because long ultrasonic excitation time causes an increase in dislocation density (after ultrasonic vibration stoppage); therefore, more dislocations tend to tangle and rearrange into the dislocation wall, which is the premise of the formation of sub-grain boundary [22]. As exhibited here by a representative TEM image, Figure 7, most of these subgrains (approx. 1 µm long) indeed contain a very high-density of dislocations. Their boundaries are mostly equilibrium, and without the Moiré contrast, which is attributed to boundaries with higher misorientation angles. The TEM observations are in good agreement with the EBSD maps, Figure 6, in which a much greater density of deformation bands was observed in the USV sample.

**Figure 8:**
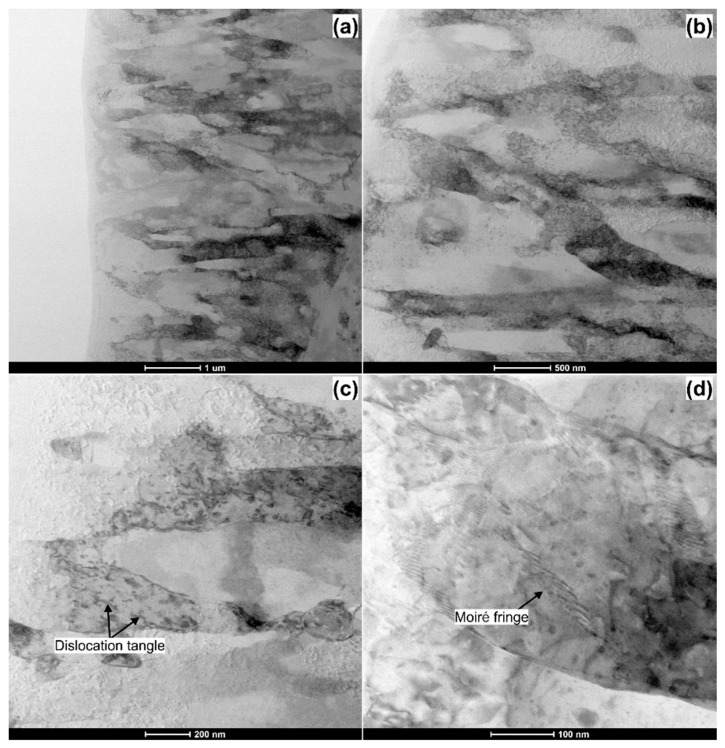
STEM bright-field images of the AlMg3 sample deformed with the action of ultrasonic vibrations. (**a**) low magnification image of the deformed area; (**b**) high magnification image of the deformed area; (**c**) magnified image of the deformed area showing a dislocation tangle; (**d**) magnified image of the deformed area showing a Moiré fringes.

Figure 8 shows the STEM (scanning transmission electron microscopy) bright-field images of the AlMg3 sample deformed by the ultrasonic vibrations. As it can be seen, the microstructure is composed of a mixture of dislocation cells and lamellar subgrains, Figure 8a,b. These subgrains are characterized by their size of about 1 µm in length and 400 nm in width. Their boundaries have diversified character. Some of them are irregular and poorly defined, indicating a high-energy non-equilibrium configuration, whereas other ones are sharp and well-defined, indicating their higher misorientation angle.

Observations at a higher magnification, Figure 8c,d, reveals multiple nanosized precipitates and dislocation tangles in these lamellar sub-grain interiors. The visible patterns in Figure 7d are Moiré fringes [42]. They form a domain structure with an average size of 100–200 nm. Their existence indicates the presence of the very low angle grain boundaries (typically with a very low misorientation angle θ < 2°). This high fraction of LAGBs in the form of Moiré boundaries does not seem to be in line with our EBSD analysis; however, some studies [43,44,45] indicate that the Moiré boundaries are not generally detected by EBSD. It is worth mentioning that the formation of Moiré fringes may be an indicator of a crystallite rotation caused by the ultrasonic vibration during the compression process [46,47]. Dutta et al. [48] suggested that plastic deformation accommodates by ultrasound-induced sub-grain boundary rotation. As these sub-boundaries reorient, there is an increase in orientation difference at the boundaries followed by a sub-boundary rotation, and the formation of grains with higher grain boundary misorientation occurs. This might be a reason why the EBSD study revealed the higher fraction of HAGBs in the sample deformed by ultrasound vibrations.

### 3.4. Flow Stress Analysis

**Figure 9:**
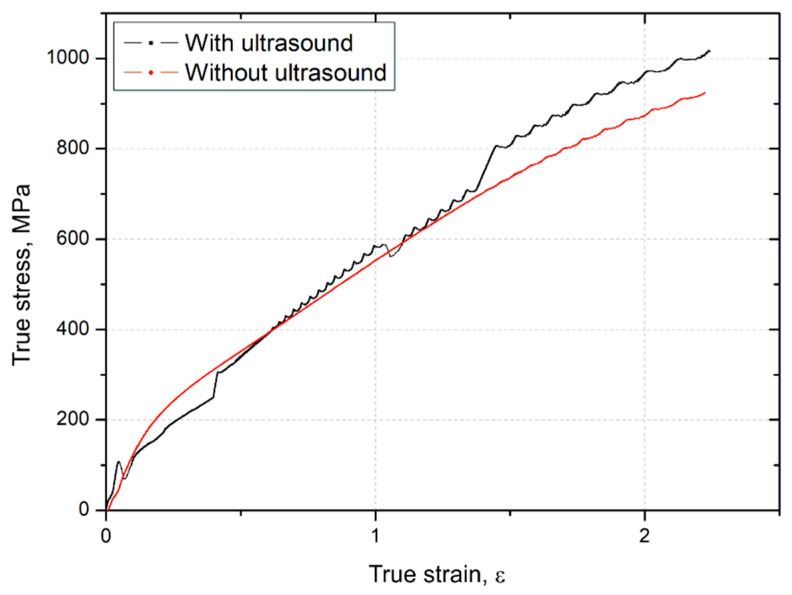
True stresses as a function of strains in the process of upsetting without USV and with the application of USV.

Figure 9 presents the relation between the stress and strain of the AlMg3 alloy deformed with (black curve) and without (red curve) an action of ultrasonic vibrations. As it can be seen from the direct comparison of both curves, the flow stress decreases immediately (from ~107 MPa to ~70 MPa) during compression when the USV is applied for the first time in this experiment (at the true strain value of ε = 0.05). This phenomenon is also known as the “Blaha effect” [44], which shows a stress reduction in the metallic materials during deformation assisted with the ultrasonic vibration. The stress reduction occurs because the ultrasound energy is absorbed by the lattice defects, such as dislocations, which can help reduce the activation energy of the dislocation movement and promote the movement of the dislocations [49,50]. Thereby, the plastic deformation is easier, and the stress is lowered. When the action of the ultrasonic vibration is removed (at the true strain value of ε = 0.4), the stress recovers to a normal level and then, after a while, to a slightly higher level (at the true strain value of ε = 0.6). At the same time, the course of the stress–strain curve changes. Typical flow serrations, which are specific signs of the PLC effect, appear. The PLC band generation generally is the result of the dislocation assembly motion and dislocation forest formation [51]. Here, this effect could be explained in terms of superposing the monotonically increasing stresses generated by the compressive deformation with the much smaller fluctuating stresses generated by the ultrasonic oscillations. The resulting vibrations make dislocation unpinning easier, thus promoting continuous propagation of the bands bringing about the PLC effect [30]. During the subsequent (second) action of ultrasonic vibrations at a higher true strain value of ε=1.1, the flow stress reduction is much lower, approx. 25 MPa, but when the USV is removed, the stress returns to a much higher value, approx. 100 MPa, which can be explained by a residual hardening effect.

### 3.5. Hardness

**Figure 10:**
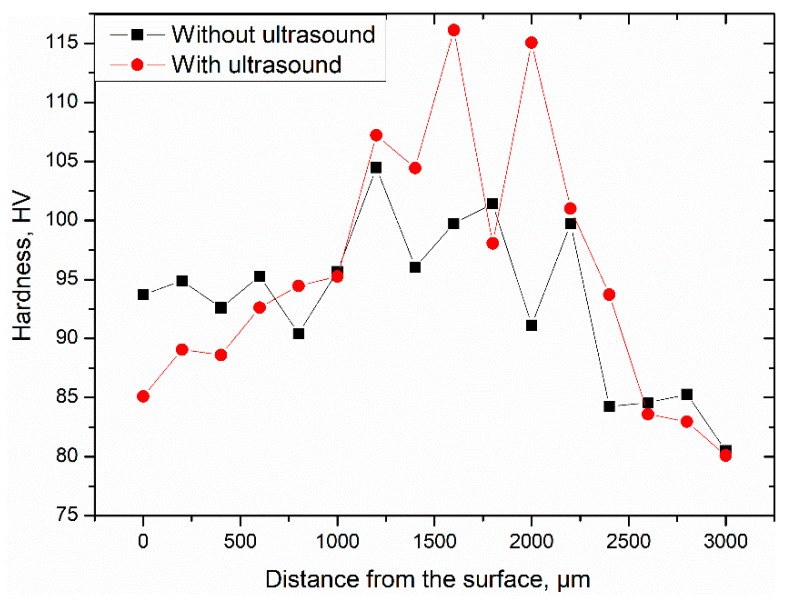
Results of Vickers microhardness measurements.

To evaluate the strength of AlMg3 aluminum samples, microhardness measurements were performed. The results plotted versus relative position across the thickness of the samples are presented in Figure 10. As expected, the non-homogeneous microstructure refinement (Figure 4a,c) resulted in nonuniform microhardness distribution. Therefore, the highest hardness values were recorded in the center of the samples, where the deformation degree is largest, while the lowest values near the top and bottom of the samples. What is more important, one can see that deformation with the action of ultrasonic vibrations resulted in a certain increase in microhardness, which is especially visible in zone II, with the highest deformation degree. This increase in strength can be attributed to the acoustic residual hardening effect that was observed from experimental results (Figure 9) as a rise up in the flow stress level as well as the grain refinement [27].

Compared to the microhardness of the initial condition sample ~68 HV, the compressed counterparts microhardness was improved to ~104 HV (without USV) and 116 HV (with USV). A very similar hardness enhancement was recorded in our earlier reported studies on the same alloy after the ECAP process [15,52].

## 4. Conclusions

This manuscript shows comparatively the differences between the microstructures and mechanical properties of the AlMg3 alloy deformed with the application of USV and without it. The major conclusions from this work can be summarized as follows:

- The application of ultrasonic vibrations may initiate the shear bands formations intensifying the grain refinement. In this study, the ultra-fine grained (UFG) AlMg3 alloy with the sub-grain size of about 1 µm was obtained;
- A temporary acoustic softening was observed after excitation of the sample by ultrasonic energy during compression test, and the residual hardening phenomenon was illustrated once the vibration was stopped off after the process;
- Ultrasonic vibrations promote continuous propagation of the bands bringing about the Portevin–Le Chatelier effect (PLC) in AlMg3 alloy during upsetting;
- The effect of ultrasonic vibration—acoustic hardening as well as an increase in dislocation density and change in microstructural characteristics—grain refinement, is closely related to the observed increase in hardness value of investigated AlMg3 aluminum alloy.

## Figures and Tables

**Table 1:** Chemical composition of the AlMg3 aluminum alloy (mass, %).

Element	Mg	Fe	Si	Cu	Ti	Al
Mass, %	3.1	0.07	0.07	0.01	0.01	balance

## Data Availability

The data presented in this study are available on request from the corresponding author.
